# Immediate memory is associated with alexithymia in Chinese Han first-episode, drug-naïve major depressive disorder

**DOI:** 10.3389/fpsyt.2025.1473204

**Published:** 2025-03-26

**Authors:** Xue Tian, Feng-feng Bai, Yong-ping Zhao, Ying Gao, Yu-ting Wang, Yuan Liu, Chu-hao Zhang, Mei-juan Li, Jie Li

**Affiliations:** ^1^ Institute of Mental Health, Tianjin Anding Hospital, Mental Health Center of Tianjin Medical University, Tianjin, China; ^2^ The National Clinical Research Center for Mental Disorders and Beijing Key Laboratory of Mental Disorders, Beijing Anding Hospital, Beijing, China

**Keywords:** alexithymia, difficulty identifying feelings, neurocognition, immediate memory, major depressive disorder

## Abstract

**Background:**

Alexithymia is defined as a difficulty in identifying and describing one’s own emotions. It represents a risk factor for cognitive deficits and is frequently observed in individuals with depressive disorders. However, the relationship between alexithymia and neurocognitive function in major depressive disorder (MDD) is still unknown. This study aimed to explore the association between alexithymia and neurocognition in patients with MDD.

**Methods:**

A total of 134 Chinese Han first-episode drug-naïve patients with MDD were recruited. The 20-item Toronto Alexithymia scale (TAS-20), the Repeatable Battery for the Assessment of Neuropsychological Status (RBANS), the 9-item Patient Health Questionnaire (PHQ-9) and the Generalized Anxiety Disorder-7 items (GAD-7) was used to assess alexithymia, neurocognitive functioning, and emotion. Multivariable liner regression models were used to estimate the association between alexithymia and neurocognition. Interaction and stratified analyses were conducted according to age, gender, marital and education status.

**Results:**

Among the 134 patients with MDD, 55 participants (41%) had alexithymia. In the fully adjusted model, TAS total score (TAS-T) (β: -0.34, 95% CI: -0.61~ -0.07) and difficulty identifying feelings (DIF) (β: -0.8, 95% CI: -1.3~-0.31) were statistically significantly associated with immediate memory.

**Conclusions:**

Higher level of alexithymia, particularly the difficulty identifying feelings facet, is associated with lower scores of immediate memory in patients with MDD.

## Introduction

Major depressive disorder (MDD) is a prevalent psychiatric disease with high rates of morbidity, disability, and mortality and has become a worldwide health concern (1). In addition to mood symptoms, individuals with depression often experience impaired cognitive functioning (2). Psychiatrists are increasingly focusing on the cognitive dysfunction of depression, preferring to view it as a stand-alone symptom rather than a secondary phenomenon to the mood symptoms of depression. The diagnostic criteria for MDD in both the Diagnostic and Statistical Manual of Mental Disorders, Fifth Edition (DSM-5) and the International Classification of Diseases, Eleventh Revision (ICD-11) focus on changes in cognitive function (3, 4). Cognitive dysfunction, which is a common symptom of depressive disorders, not only restricts the patient’s ability to perform in multiple areas, but also significantly degrades their overall quality of life. Additionally, it increases the risk of physical illnesses and serves as a factor that negatively impacts the prognosis of depression (5). Therefore, cognitive impairment in patients with depressive disorders is a noteworthy clinical target for attention.

Alexithymia is a deficit in the cognitive processing of emotions with three major facets, (1) difficulty in identifying one’s feelings and distinguishing them from bodily sensations; (2) difficulty in describing one’s feelings to others; and (3) an externally oriented cognitive style (6). Alexithymia frequently occurs in a variety of somatic and psychiatric disorders (7–12), however, individuals suffering from depression have a higher rate of alexithymia than people with other psychiatric disorders (13). Numerous studies have found that disturbances in neurocognitive functioning are related to the degree of alexithymia (11, 14). Studies in different populations, both healthy and clinical individuals, have found that alexithymia is associated with multiple dimensions of neurocognitive functioning, including language, executive and visuospatial abilities, attention and memory (15, 16). Neuroimaging studies further support the idea that alexithymia can have an impact on cognitive function (17).

Despite the association between alexithymia and depression, as well as cognitive functioning, to the best of our knowledge, no study has investigated the relationship between alexithymia and neurocognitive function in patients with depression. This study aimed to explore the association between alexithymia and neurocognitive function in patients with MDD. Given that neurocognitive function and alexithymia may be influenced by medication exposure and disease progression, only patients with the first episode, drug-naïve MDD were included in the study (18). Because both alexithymia and cognitive functioning were culturally influenced, only the Han Chinese, the largest population in China, were selected for this study (19, 20). Since there were a limited number of studies in this area, our study was exploratory and with no specific hypotheses on which particular domain of neurocognitive function was specifically associated with alexithymia in patients with MDD.

## Materials and methods

### Study design and study population

We conducted this cross-sectional study to explore the association between alexithymia and neurocognition in patients with MDD, following the guidelines of the STROBE statement. Data was collected between January 2021 and July 2022 in China. The study comprised of 134 drug-naïve patients with their first-episode depression. These participants were selected from Tianjin Anding Hospital. The inclusion criteria were as follows: (1) First diagnosed with MDD according to DSM-5; (2) All patients were first-episode without previous pharmacological treatment; (3) aged 18-60 years. Participants were excluded based on the following criteria: (1) ethnic background other than Han; (2) serious physical disease; (3) a history of any mental illnesses other than MDD; (4) pregnancy; and (5) substance abuse. The ethics committee of the Tianjin Anding Hospital approved the study, which was conducted in accordance with the Declaration of Helsinki. All participants signed a written informed consent before assessment.

### Data collection

#### Alexithymia

The 20-item Toronto Alexithymia Scale (TAS-20) was used to measure alexithymia (21). It has demonstrated favorable internal consistency and test-retest reliability in the general population (22), with Cronbach α-coefficients ranging from 0.81 to 0.86 (21). It consists of three dimensions: difficulty in identifying feelings (DIF), difficulty in describing feelings (DDF), and externally oriented thinking (EOT). Each item on the TAS-20 is scored on a five-point Likert, with total scores ranging from 20 to 100. Higher total scores reflect greater alexithymia. Individuals who score 61 or above on the TAS total score (TAS-T) are classified as having alexithymia (23).

#### Neurocognition

The Repeatable Battery for the Assessment of Neuropsychological Status (RBANS) was used to evaluate neurocognitive functions (24). The RBANS is a widely used neuropsychological battery which assesses different domains of cognitive function, including Attention, Language, Visuospatial/construction, Immediate memory, and Delayed memory. RBANS has good validity and reliability in Chinese people (25). The RBANS total score demonstrated strong internal consistency, with a reliability coefficient of 0.806. The Cronbach’s α values for each of the RBANS subscales ranged from 0.142 to 0.727 (25).

#### Psychological assessment

The Patient Health Questionnaire-9 (PHQ-9) was used to assess the severity of depression in the last two weeks. It is a self-rated measure of depression consisting of nine items that align with the criteria for major depression in the Diagnostic and Statistical Manual of Mental Disorders, Fourth Edition (DSM-IV) (26). PHQ-9 scores range from 0 to 27, with higher scores indicating more severe depression. Depressive symptoms were categorized by severity into five groups: minimal (scores of 0-4), mild (5-9), moderate (10-14), moderately severe (15-19), and severe (20-27) (27). It has been demonstrated as a valid and reliable measure of depression among the Chinese population, with Cronbach α-coefficients ranging from 0.84 to 0.87 (27).

The Generalized Anxiety Disorder-7 items (GAD-7) was used to evaluate the severity of anxiety. GAD-7 is developed by Spitzer and his colleagues to evaluate the severity of anxiety in the last two weeks (28). It is a 7-item self-report scale with a range of scores from 0 to 21, with higher scores indicating greater anxiety severity. It has been discovered to be a reliable and valid tool for screening GAD, as demonstrated in a Chinese population (29). The Cronbach’s α coefficient was 0.898, indicating excellent internal consistency (29).

Demographic characteristics (age, gender, marital status, and education) were recorded through a standardized questionnaire. Alexithymia and emotion were assessed by the patients themselves after a detailed explanation by the psychiatrist. Two experienced and trained psychiatrists assessed neurocognitive functions using the RBANS assessment manual. The inter-rater correlation coefficient for the RBANS total score was 0.86.

### Statistical analysis

Continuous variables were presented as means and standard deviations (SD) for normal distribution, mediation and interquartile range (IQR) for non-normal distribution and categorical variables as frequencies and percentages. Differences between groups were analyzed using the student t-test for normally distributed variables, the Mann-Whitney test for skewed distribution variables, and the chi-square test for categorical variables. Multivariable liner regression models were used to assess the association of alexithymia and neurocognition. Unadjusted and adjusted β with 95% confidence intervals (CIs) were calculated. Model 1 was unadjusted. Model 2 was adjusted for age, gender, marital and education status. Model 3 was further adjusted for PHQ-9 and GAD-7. Given that 29 years was the mean age of the study population and that having ≥16 years of education is indicative of higher education, we conducted interaction and stratified analyses based on age grouping (above and below 29 years), gender, marital status (single and married), and education (above and below 16 years). The *P*-value reported was two-sided and statistical significance was defined as a value less than 0.05. All the analyses were performed with the statistical software packages R (http://www.R-project.org, The R Foundation) and Free Statistics software versions 1.8 (Beijing Free Kelin Medical Technology Co, Ltd.).

## Results

### Basic characteristics of the study participants

**Figure 1:**
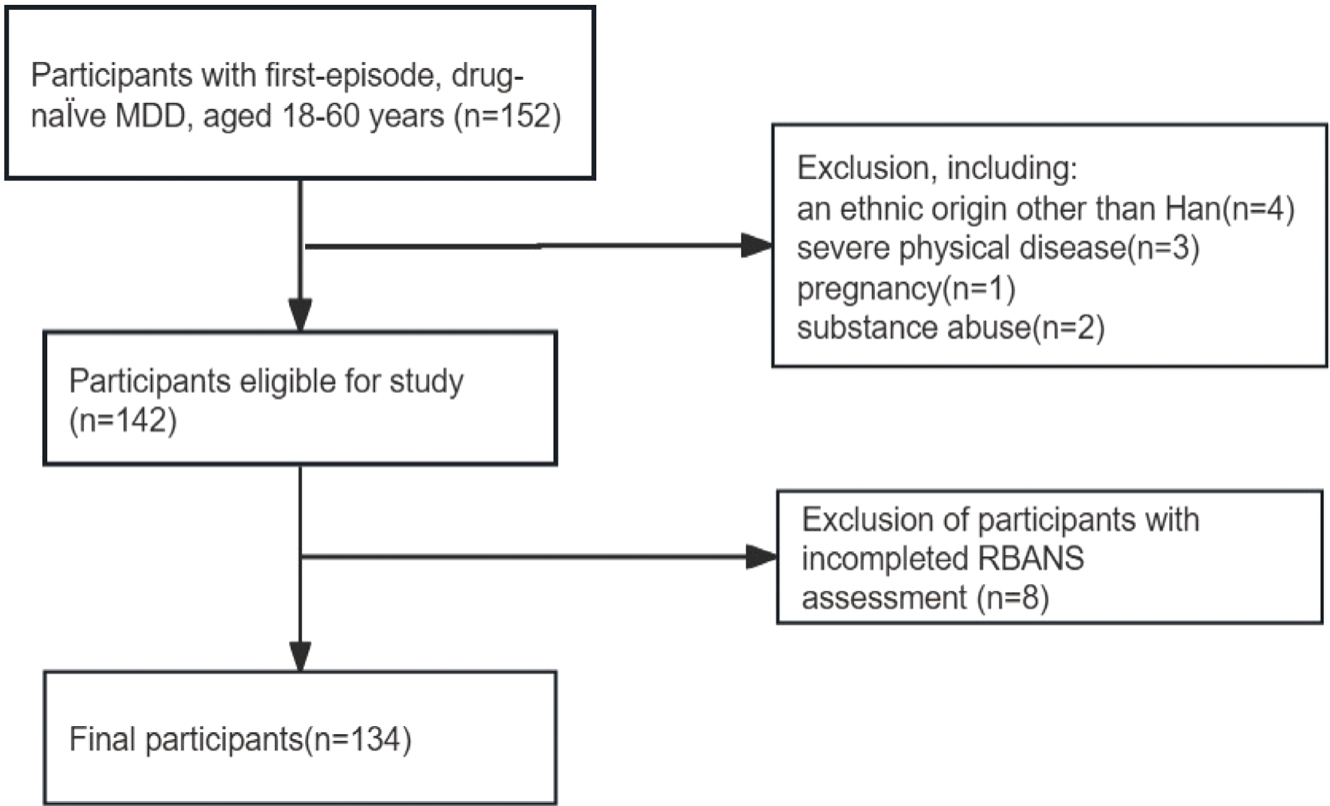
Flowchart of participant selection. MDD, major depressive disorder; RBANS, Repeatable Battery for the Assessment of Neuropsychological Status.

This study screened 152 patients with first-episode, drug-naïve MDD, of whom 10 were not included for meeting one of the exclusion criteria. Participants who failed to complete the RBANS assessment (*n*=8) were also excluded. Ultimately, 134 participants were included in the study analysis. **Figure 1** presents a flow diagram.

Finally, 134 patients were enrolled in this study, and 55 participants (41%) had alexithymia (TAS-T≥61). The average age of the participants was 29.1 (10.1) years. Of the participants, 54 (40.3%) were male, 84 (62.7%) were single, and 64 (47.8%) had higher education (years of education≥16). All participants had PHQ-9 scores of ≥5 (The percentages of different severity levels are detailed in **Supplementary Figure 1**). Individuals with alexithymia were younger (mean age, 25.6 vs. 31.4 years), more inclined to be single (41 [74.5%] vs. 43 [54.4%]) and had higher PHQ-9 scores. Additionally, a higher proportion of individuals with alexithymia had more severe depression (48 [87.3%] vs. 57 [72.2%]). **Table 1** shows the main characteristics of the study subjects.

**Table 1:** Basic characteristics of the study participants.

Variables	Total (n = 134)	Non-alexithymic (n = 79)	Alexithymic (n = 55)	p
Gender, n (%)				0.31
Male	54 (40.3)	29 (36.7)	25 (45.5)	
Female	80 (59.7)	50 (63.3)	30 (54.5)	
Marital Status, n (%)				0.018
Single	84 (62.7)	43 (54.4)	41 (74.5)	
Married	50 (37.3)	36 (45.6)	14 (25.5)	
Education, year, n (%)				0.797
<16	70 (52.2)	42 (53.2)	28 (50.9)	
≥16	64 (47.8)	37 (46.8)	27 (49.1)	
Depression severity, n (%)				0.037
mild-moderate	29 (21.6)	22 (27.8)	7 (12.7)	
moderately severe- severe	105 (78.4)	57 (72.2)	48 (87.3)	
Age, year	29.1 ± 10.1	31.4 ± 10.9	25.6 ± 7.6	< 0.001
TAS total score	58.5 ± 9.9	51.8 ± 6.3	68.1 ± 5.2	< 0.001
DIF	22.0 ± 5.5	18.7 ± 3.7	26.7 ± 4.1	< 0.001
DDF	16.0 ± 3.3	14.2 ± 2.5	18.7 ± 2.3	< 0.001
EOT	20.5 ± 3.6	18.9 ± 3.3	22.7 ± 2.7	< 0.001
RBANS total score	91.9 ± 14.9	92.7 ± 15.3	90.8 ± 14.5	0.485
Attention	109.7 ± 14.5	110.9 ± 13.1	108.1 ± 16.3	0.285
Language	95.3 ± 14.0	96.2 ± 14.5	94.1 ± 13.2	0.388
Visuospatial	88.7 ± 17.7	88.4 ± 17.8	89.1 ± 17.8	0.817
Immediate Memory	85.9 ± 15.4	87.3 ± 16.3	84.0 ± 14.1	0.232
Delayed Memory	91.1 ± 14.1	91.3 ± 15.0	90.8 ± 12.9	0.867
PHQ-9	18.2 ± 5.0	17.3 ± 5.3	19.4 ± 4.4	0.017
GAD-7	13.0 ± 5.0	12.5 ± 5.2	13.7 ± 4.6	0.159

TAS, Toronto Alexithymia scale; DIF, difficulty identifying feelings; DDF, difficulty describing feelings; EOT, externally oriented thinking; RBANS, Repeatable Battery for the Assessment of Neuropsychological Status; PHQ-9, Patient Health Questionnaire-9 items; GAD-7, Generalized Anxiety Disorder-7 items.

The severity of depression was categorized based on PHQ-9 total scores:mild (5-9), moderate (10-14), moderately severe (15-19), and severe (20-27).

### Associations between alexithymia and neurocognition

The relationship between alexithymia and neurocognition is presented in **Table 2**. The univariate linear regression analysis showed significant negative associations between TAS-T and language (β: -0.25, 95% CI: -0.49~-0.02) as well as immediate memory function (β: -0.3, 95% CI: -0.57~ -0.04). Additionally, there were negative associations between the DIF subscale and language (β: -0.49, 95% CI: -0.93~-0.06) and immediate memory function (β: -0.64, 95% CI: -1.12~-0.16).

**Table 2:** Multivariable liner analysis evaluating the association between alexithymia and cognitive score.

Variable	Attention		Language		Visuospatial		Immediate Memory		Delayed Memory	
	β (95% CI)	P	β (95% CI)	P	β (95% CI)	P	β (95% CI)	P	β (95% CI)	P
	Model 1		Model 1		Model 1		Model 1		Model 1	
TAS total	-0.16 (-0.41~0.1)	0.22	-0.25 (-0.49~-0.02)	0.04*	-0.1 (-0.41~0.21)	0.54	-0.3 (-0.57~-0.04)	0.03*	-0.14 (-0.39~0.1)	0.25
DIF	-0.36 (-0.82~0.1)	0.12	-0.49 (-0.93~-0.06)	0.03*	-0.25 (-0.82~0.31)	0.38	-0.64 (-1.12~-0.16)	0.01^*^	-0.29 (-0.74~0.16)	0.20
DDF	-0.35 (-1.12~0.43)	0.38	-0.57 (-1.31~0.17)	0.13	-0.3 (-1.25~0.64)	0.53	-0.64 (-1.46~0.17)	0.12	-0.09 (-0.85~0.66)	0.81
EOT	-0.08 (-0.78~0.62)	0.82	-0.34 (-1.01~0.33)	0.31	0.09 (-0.77~0.94)	0.84	-0.33 (-1.07~0.41)	0.38	-0.35 (-1.03~0.33)	0.31

	Model 2		Model 2		Model 2		Model 2		Model 2	
TAS total	-0.14 (-0.41~0.12)	0.29	-0.2 (-0.46~0.05)	0.12	-0.09 (-0.4~0.23)	0.59	-0.33 (-0.59~-0.07)	0.02*	-0.13 (-0.38~0.12)	0.30
DIF	-0.33 (-0.82~0.16)	0.19	-0.4 (-0.87~0.07)	0.10	-0.28 (-0.87~0.31)	0.35	-0.77 (-1.25~-0.29)	0.002*	-0.3 (-0.76~0.16)	0.21
DDF	-0.31 (-1.11~0.5)	0.46	-0.38 (-1.16~0.4)	0.34	-0.25 (-1.22~0.73)	0.62	-0.7 (-1.51~0.12)	0.10	-0.01 (-0.77~0.76)	0.98
EOT	-0.1 (-0.79~0.59)	0.78	-0.32 (-0.99~0.35)	0.35	0.14 (-0.69~0.98)	0.74	-0.24 (-0.94~0.46)	0.51	-0.3 (-0.95~0.35)	0.37

	Model 3		Model 3		Model 3		Model 3		Model 3	
TAS total	-0.13 (-0.4~0.14)	0.36	-0.23 (-0.49~0.03)	0.09	-0.13 (-0.45~0.2)	0.45	-0.34 (-0.61~-0.07)	0.01*	-0.11 (-0.37~0.14)	0.38
DIF	-0.31 (-0.82~0.2)	0.24	-0.47 (-0.96~0.02)	0.07	-0.38 (-1~0.23)	0.23	-0.8 (-1.3~-0.31)	0.002*	-0.26 (-0.73~0.22)	0.30
DDF	-0.28 (-1.1~0.54)	0.50	-0.4 (-1.19~0.39)	0.32	-0.29 (-1.28~0.69)	0.56	-0.7 (-1.52~0.12)	0.10	0.02 (-0.75~0.78)	0.96
EOT	-0.06 (-0.76~0.64)	0.87	-0.35 (-1.03~0.32)	0.31	0.09 (-0.76~0.93)	0.84	-0.26 (-0.96~0.45)	0.48	-0.3 (-0.95~0.36)	0.38

Modal 1: No adjustment.

Modal 2: Adjusted for gender, age, marital status and education.

Modal 3: Adjusted for gender, age, marital status, education, PHQ-9 and GAD-7.

TAS, Toronto Alexithymia scale; TAST, TAS total score; DIF, difficulty identifying feelings; DDF, difficulty describing feelings; EOT, externally oriented thinking; PHQ-9, Patient Health Questionnaire-9 items; GAD-7, Generalized Anxiety Disorder-7 items; CI, confidence interval.

*P<0.05.

After adjusting for gender, age, marital status and education (Model 2), the associations between TAS-T (β: -0.2, 95% CI: -0.46~0.05) and language and between DIF (β: -0.4, 95% CI: -0.87~0.07) and language disappeared. Nevertheless, the negative association between TAS-T and immediate memory and between DIF and immediate memory were still significant in both Model 2 and Model 3. Model 3 was further adjusted for PHQ-9 and GAD-7. In fully adjusted Model 3, the immediate memory score decreased by 0.34 points for each 1-point increase in the total TAS score (Model 3, β: -0.34, 95% CI: -0.61~ -0.07) and decreased by 0.8 points for each 1-point increase in the DIF subscale score (Model 3, β: -0.8, 95% CI: -1.3~-0.31).

### Subgroup analyses

**Figure 2:**
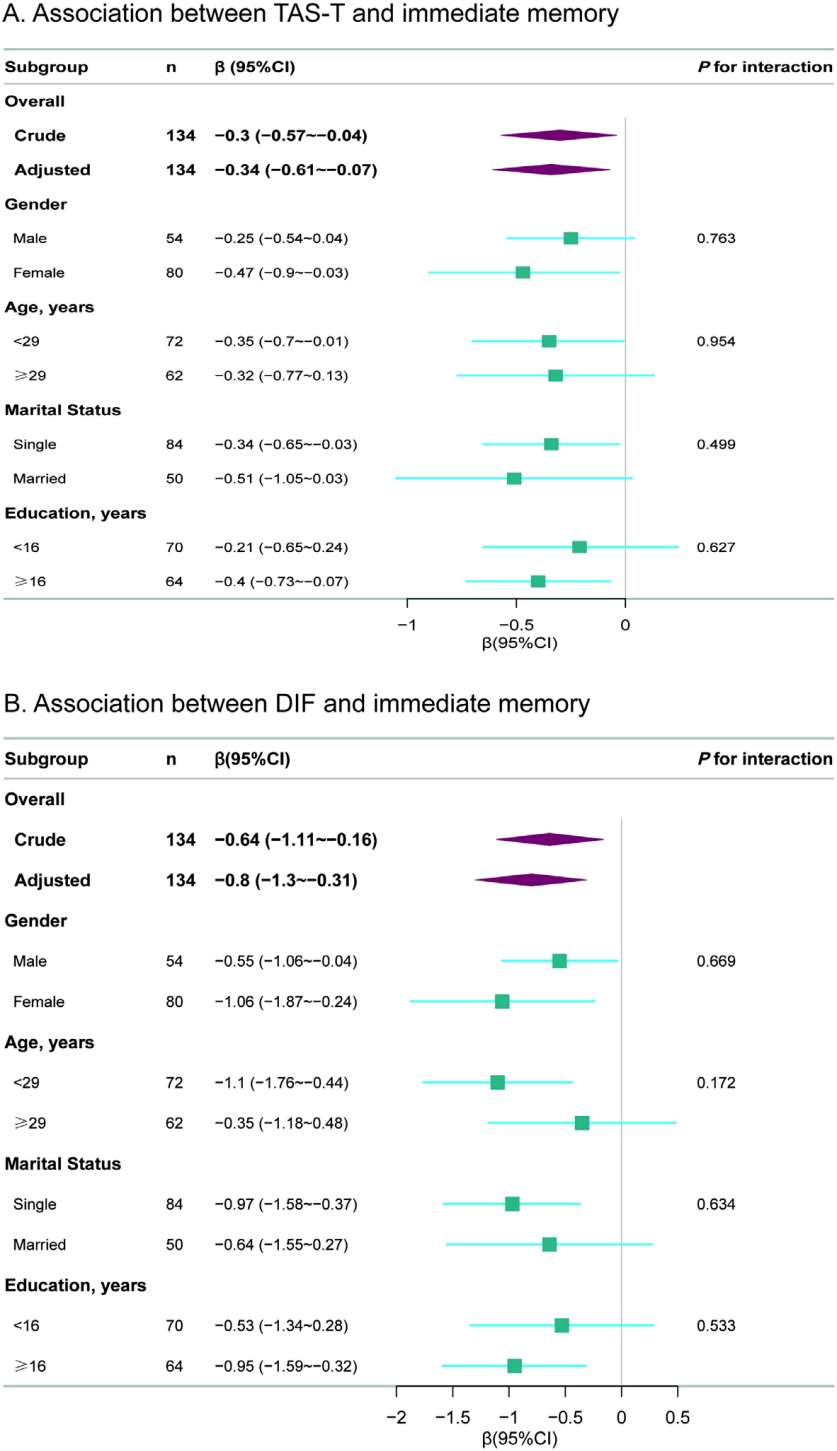
Subgroup analyses for the associations of TAS total and DIF subscale scores with immediate memory. **(A)** Association between TAS-T and immediate memory **(B)** Association between DIF and immediate memory. TAS, Toronto Alexithymia scale; TAS-T, TAS total score; DIF, difficulty identifying feelings; CI, confidence interval. The *p*-value for interaction represents the likelihood of interaction between the variable and the TAS total score or the DIF subscale score. Each stratification was adjusted for gender, age, education, marital status, PHQ-9 and GAD-7.

**Figures 2A, B** displays the results of the stratified and interaction analyses investigating the associations between the TAS total score and immediate memory, as well as between the DIF score and immediate memory. In the stratified analysis, results were consistent with those observed in the multivariable linear regression analysis. No significant interactions were found within any subgroups, including gender, age, marital status and education level (all *P* for interaction>0.05).

## Discussion

This cross-sectional study examined the association between alexithymia and neurocognitive function in Chinese Han first-episode, drug-naïve MDD. The results revealed a negative association between alexithymia and immediate memory. This negative association remained significant after adjusting for covariates including age, gender, marital and education status and emotion and remained stable across subgroups. This suggests that level of alexithymia was negatively associated with immediate memory independent of demographic factors and emotions in first-episode, drug-naïve MDD. Interestingly, when comparing individuals with and without alexithymia, we did not observe a significant difference in immediate memory performance. The reasons may be as follows: First, the immediate memory performance of the alexithymia group was indeed lower than that of the non-alexithymia group. However, the relatively small sample size limited the ability to detect significant differences. Second, we observed a negative association between alexithymia as a continuous variable and immediate memory. This association may not be significant when alexithymia is treated as a categorical variable because it reduces statistical power. Third, the alexithymia group had a relatively younger age distribution and a higher proportion of participants with higher education. These demographic characteristics may have influenced memory performance, thereby masking the true relationship between alexithymia and immediate memory. In contrast, our regression and stratified analyses accounted for multiple covariates and potential confounders, which allowed us to more accurately identify the independent impact of alexithymia on memory performance.

Studies have shown that alexithymia is associated with impaired cognitive processing of emotional information and with deficits in learning and memory. This is particularly the case when it comes to emotional information and contexts (30). A small sample of older adults reported a negative correlation between story and figure recall and alexithymia (31). Similarly, in a study conducted on young adults, it was found that those with high levels of alexithymia had a decreased immediate recall ability but unimpaired long-term recognition of the words for neutral memoranda in a neutral context (32). Vermeulen found that alexithymia inhibited short-term memory but not long-term retrieval, regardless of whether the information was emotional or not (33). Conversely, some studies have found that individuals with higher levels of alexithymia may exhibit preserved or even improved memory for emotionally provocative words (34). Recent studies have explored the relationship between alexithymia and neurocognitive function across various patient populations. However, the specific associations between alexithymia and cognitive function vary across diseases, likely attributable to differences in disease pathology, heterogeneity of cognitive tests used, and sample characteristics. For instance, a recent meta-analysis found that the prevalence of alexithymia in Parkinson’s disease (PD) is significantly higher than in the general population and is associated with cognitive impairment (35). Studies on PD have shown that alexithymia correlates with deficits in the visuospatial domain and executive function, rather than memory functions (36). Similar findings were observed in HIV patients, where alexithymia correlated with deficits in visuospatial and executive functions but not memory impairments (37). A meta-analysis of eight studies revealed a moderate to large effect size for the association between alexithymia and schizophrenia (38). In schizophrenia, the EOT dimension of alexithymia has been linked to deficits in working memory (11, 39). In contrast, study on multiple sclerosis (MS) found that alexithymia correlated with depression and anxiety but not with cognition (40), suggesting that its primary impact on emotional regulation rather than cognition. Our study revealed a statistically significant negative association between level of alexithymia and immediate memory in patients with MDD. These findings underscore the significant role of population-specific factors in shaping the relationship between alexithymia and cognitive function. Further investigations across diverse populations are warranted to provide a more comprehensive understanding of this complex relationship.

Research has shown that alexithymia, which refers to the difficulty in identifying and describing one’s own emotions, is related to decreased empathic behaviors (41, 42). Alexithymia appeared to underlie difficulties in key cognitive processes essential for empathy, specifically in sharing the emotional state of others (43). This lack of empathy results from difficulties in perceiving emotions, leading to misinterpretation of social cues and ultimately resulting in a lack of understanding of others’ emotions (44). Studies have shown that individuals suffering from MDD experience diminished feelings of compassion (45). Furthermore, recent evidence has shown that the presence of alexithymia contributes to a deficit in empathy in depression (46). Previous literature has suggested that the motivation to empathize with others can guide attention and memory (47). Although the underlying mechanism cannot be tested in the present study, it is possible that the ability of empathy to raise emotional arousal can enhance the encoding of information (48). Consequently, “DIF”, a core feature of alexithymia in patients with major depressive disorder (MDD), may be significantly associated with memory deficits, potentially mediated by reduced empathic ability.

Alexithymia, an impairment of affective and cognitive emotional processing, may reflect changes in brain regions important for cognitive function. A study on general population sample showed that the TAS-20 total score and DIF were both associated with less gray matter volumes of bilateral dorsal anterior cingulate cortex (ACC) (49). ACC is an essential area of the cerebral cortex for cognitive deficits production (5, 50). Recent evidence has shown that the human anterior cingulate and orbitofrontal cortex regions are linked to Meynert’ septal nuclei and basal forebrain nucleus, which contain cholinergic neurons that project to the hippocampus and neocortex respectively (51). The impairment of the ACC system has been proposed as a cause for hippocampal episodic memory storage impairment (51). Additionally, volume reductions in the ACC have been observed in early-onset mood disorders with some specificity (52). Alexithymia is also strongly related to chronic stress, which is associated with brain structural and neuroendocrine alterations which may act as a mechanism for deficits in memory function (16). In particular, brain regions such as the hippocampus and prefrontal cortex, which are crucial for declarative memory functioning, especially short-term memory, have each been found to be affected by chronic stress (53, 54). Overall, further research is needed to understand the mechanisms underlying the association between alexithymia and memory impairment in patients with MDD, including neuroimaging and basic science research.

The expression and experience of emotions are inherently influenced by culture (19). For example, Western cultures encourage the expression of emotions more than Eastern cultures. Given that alexithymia is primarily characterized by difficulties in emotion identification and communication, it is influenced by culture (55). Previous research has shown that Asian groups exhibit higher levels of alexithymia compared to their European American counterparts (55). Moreover, different cultural backgrounds can exert a significant impact on specific neurocognitive processes (20), such as attention (56) and memory (57). Research in the field of neuroscience has also confirmed these findings (58, 59). Different cultural environments and diverse patterns of social interaction can profoundly influence memory development (60). Given the influence of culture on alexithymia and cognitive functioning, the present study chose a specific population, the Han Chinese, as the subject of the study and found negative associations between alexithymia and immediate memory in this population. Nevertheless, future studies with larger sample sizes and diverse populations are needed to further clarify the association between alexithymia and cognitive functioning in different cultural contexts.

In this study, we observed a negative association between alexithymia and verbal function in the unadjusted model. However, this association was no longer significant after adjusting for covariates. This finding aligns with the alexithymia–language hypothesis proposed by Hobson, which suggests that language deficits lead to impaired emotional awareness and, consequently, alexithymia (61). Studies in both clinical and healthy populations have supported this hypothesis, indicating that higher levels of alexithymia are associated with poorer verbal function (62, 63). The language difficulties observed in alexithymia may reflect a lack of early social language learning opportunities. Longitudinal studies have also demonstrated that language development in childhood can influence the manifestation of alexithymia in adolescence (64). However, some studies have reached the opposite conclusion (65). Given the considerable heterogeneity in sample characteristics, clinical presentations, and methodologies across studies, the relationship between alexithymia and language remains uncertain (66). According to the language hypothesis, individuals with language deficits are more likely to exhibit higher levels of alexithymia compared to those with normal language function. However, not all individuals with alexithymia exhibit significant language impairments (60). This may explain why the association between alexithymia and verbal function was attenuated after controlling for various demographic characteristics in our study.

### Limitations

There are several limitations to our study that need to be acknowledged. Firstly, owing to the cross-sectional design of our study, it is impossible to establish any causality between the variables. Secondly, although our sample size is not small when compared to previous studies in this area, it still remains a limiting factor of our research. Thirdly, the emotion scales we used in our study, PHQ-9 and GAD-7, are self-assessment questionnaires. However, emotion evaluation was used as a covariate in this study to test the stability of the results. In future studies if emotion seen as an observed variable, including other assessment scales such as Hamilton Rating Scale for Depression (HAMD) and Hamilton Anxiety Rating Scale (HAMA) would provide a more comprehensive evaluation of emotions. Fourthly, despite the application of regression models and stratified analyses, complete elimination of residual confounding effects stemming from unmeasured or unknown factors cannot be guaranteed. For instance, there may be other psychological variables that might influence cognitive processes or the relationship between alexithymia and memory deficits. Fifthly, our study population included individuals with varying severities of depression, which may have influenced the association between alexithymia and cognitive function. Future studies should consider stratifying analyses by depression severity to better understand these complex relationships. Sixthly, it may be difficult to generalize the findings to the entire population of depressed patients because alexithymia is closely related to culture, and only Chinese Han patients were selected in this study. Furthermore, different subtypes of MDD may exert distinct impacts on cognitive function (67). Our study did not investigate the comorbid symptoms of depression to differentiate between subtypes. Therefore, it would be an interesting direction for future research to explore the relationship between alexithymia and cognitive function across different subtypes of MDD. Nevertheless, our study represents the first attempt to explore the relationship between alexithymia and neurocognition in patients with MDD, which could contribute to a deeper understanding of the cognitive impairment in MDD. Despite these limitations, future research should focus on well-designed and larger sample longitudinal studies to validate our findings.

## Conclusions

In conclusion, higher level of alexithymia, particularly the difficulty identifying feelings facet, is associated with lower immediate memory scores among Chinese Han population with depression. More attention should be paid to altered cognitive functioning in patients with MDD who suffer from alexithymia, especially those with difficulty in identifying feelings. Future studies would further explore the mechanisms of the association between alexithymia and neurocognitive functioning.

## Data Availability

The raw data supporting the conclusions of this article will be made available by the authors, without undue reservation.

## Supplementary material

Supplementary Figure 1 Percentages of different depression severity levels according to PHQ-9 total score.

## References

[1] Yao JY Zheng ZW Zhang Y Su SS Wang Y Tao J. Electrophysiological evidence for the characteristics of implicit self-schema and other-schema in patients with major depressive disorder: An event-related potential study. Front Psychiatry. (2023) 14:1131275. doi: 10.3389/fpsyt.2023.1131275 37113549 PMC10126260

[2] Rock PL Roiser JP Riedel WJ Blackwell AD. Cognitive impairment in depression: a systematic review and meta-analysis. Psychol Med. (2014) 44:2029–40. doi: 10.1017/s0033291713002535 24168753

[3] American Psychiatric Association. Diagnostic and Statistical Manual of Mental Disorders, Fifth Edition, Text Revision. Washington, DC: American Psychiatric Association (2022).

[4] World Health Organization. International classification of diseases (2019). Available online at: https://icd.who.int/browse/2024-01/mms/en76398729 (Accessed May 21, 2023).

[5] Czerwińska A Pawłowski T. Cognitive dysfunctions in depression - significance, description and treatment prospects. Psychiatr Pol. (2020) 54:453–66. doi: 10.12740/PP/OnlineFirst/105415 33038880

[6] Taylor GJ Bagby RM Parker JDA. What’s in the name ‘alexithymia’? A commentary on “Affective agnosia: Expansion of the alexithymia construct and a new opportunity to integrate and extend Freud’s legacy. Neurosci Biobehav Rev. (2016) 68:1006–20. doi: 10.1016/j.neubiorev.2016.05.025 27235080

[7] Carrozzino D Porcelli P. Alexithymia in gastroenterology and hepatology: A systematic review. Front Psychol. (2018) 9:470. doi: 10.3389/fpsyg.2018.00470 29681874 PMC5897673

[8] Di Tella M Benfante A Airale L Castelli L Milan A. Alexithymia and hypertension: does personality matter? A systematic review and meta-analysis. Curr Cardiol Rep. (2023) 25:711–24. doi: 10.1007/s11886-023-01894-7 PMC10307708 37212924

[9] Westwood H Kerr-Gaffney J Stahl D Tchanturia K. Alexithymia in eating disorders: Systematic review and meta-analyses of studies using the Toronto Alexithymia Scale. J Psychosom Res. (2017) 99:66–81. doi: 10.1016/j.jpsychores.2017.06.007 28712432 PMC5986724

[10] Honkalampi K Jokela M Lehto SM Kivimäki M Virtanen M. Association between alexithymia and substance use: A systematic review and meta-analysis. Scand J Psychol. (2022) 63:427–38. doi: 10.1111/sjop.12821 PMC9790486 35436351

[11] Gawęda Ł Krężołek M. Cognitive mechanisms of alexithymia in schizophrenia: Investigating the role of basic neurocognitive functioning and cognitive biases. Psychiatry Res. (2019) 271:573–80. doi: 10.1016/j.psychres.2018.12.023 30554105

[12] Benfante A Romeo A. Alexithymia among people living with HIV: A scoping review. AIDS Behav. (2023) 27:1926–41. doi: 10.1007/s10461-022-03926-9 36367612

[13] Li S Zhang B Guo Y Zhang J. The association between alexithymia as assessed by the 20-item Toronto Alexithymia Scale and depression: A meta-analysis. Psychiatry Res. (2015) 227:1–9. doi: 10.1016/j.psychres.2015.02.006 25769520

[14] Ciccarelli N Baldonero E Milanini B Fabbiani M Cauda R Di Giambenedetto S. Cognitive impairment and cardiovascular disease related to alexithymia in a well-controlled HIV-infected population. Infez Med. (2019) 27:274–82. 31545771

[15] Luminet O Nielson KA Ridout N. Having no words for feelings: alexithymia as a fundamental personality dimension at the interface of cognition and emotion. Cognit Emot. (2021) 35:435–48. doi: 10.1080/02699931.2021.1916442 33900884

[16] Terock J van der Auwera S Janowitz D Klinger-König J Schmidt CO Freyberger HJ. The relation of alexithymia, chronic perceived stress and declarative memory performance: Results from the general population. Psychiatry Res. (2019) 271:405–11. doi: 10.1016/j.psychres.2018.12.024 30530059

[17] Wingbermühle E Theunissen H Verhoeven WM Kessels RP Egger JI. The neurocognition of alexithymia: evidence from neuropsychological and neuroimaging studies. Acta Neuropsychiatr. (2012) 24:67–80. doi: 10.1111/j.1601-5215.2011.00613.x 26952949

[18] Lee RS Hermens DF Porter MA Redoblado-Hodge MA. A meta-analysis of cognitive deficits in first-episode Major Depressive Disorder. J Affect Disord. (2012) 140:113–24. doi: 10.1016/j.jad.2011.10.023 22088608

[19] Sagar R Talwar S Desai G Chaturvedi SK. Relationship between alexithymia and depression: A narrative review. Indian J Psychiatry. (2021) 63:127–33. doi: 10.4103/psychiatry.IndianJPsychiatry_738_19 PMC8214133 34194055

[20] Muggleton NG Banissy MJ. Culture and cognition. Cognit Neurosci. (2014) 5:1–2. doi: 10.1080/17588928.2014.885781 24499407

[21] Bagby RM Parker JD Taylor GJ. The twenty-item Toronto Alexithymia Scale–I. Item selection and cross-validation of the factor structure. J Psychosom Res. (1994) 38:23–32. doi: 10.1016/0022-3999(94)90005-1 8126686

[22] Zhu X Yi J Yao S Ryder AG Taylor GJ Bagby RM. Cross-cultural validation of a Chinese translation of the 20-item Toronto Alexithymia Scale. Compr Psychiatry. (2007) 48:489–96. doi: 10.1016/j.comppsych.2007.04.007 17707259

[23] Taylor GJ Bagby RM Parker JD. Disorders of affect regulation: Alexithymia in medical and psychiatric illness. Cambridge: Cambridge University Press (1997).

[24] Randolph C Tierney MC Mohr E Chase TN. The Repeatable Battery for the Assessment of Neuropsychological Status (RBANS): preliminary clinical validity. J Clin Exp Neuropsychol. (1998) 20:310–9. doi: 10.1076/jcen.20.3.310.823 9845158

[25] Cheng Y Wu W Wang J Feng W Wu X Li C. Reliability and validity of the Repeatable Battery for the Assessment of Neuropsychological Status in community-dwelling elderly. Arch Med Sci. (2011) 7:850–7. doi: 10.5114/aoms.2011.25561 PMC3258798 22291831

[26] Kroenke K Spitzer RL Williams JB. The PHQ-9: validity of a brief depression severity measure. J Gen Intern Med. (2001) 16:606–13. doi: 10.1046/j.1525-1497.2001.016009606.x PMC1495268 11556941

[27] Leung DYP Mak YW Leung SF Chiang VCL Loke AY. Measurement invariances of the PHQ-9 across gender and age groups in Chinese adolescents. Asia Pac Psychiatry. (2020) 12:e12381. doi: 10.1111/appy.12381 32011101 PMC7507123

[28] Spitzer RL Kroenke K Williams JB Löwe B. A brief measure for assessing generalized anxiety disorder: the GAD-7. Arch Intern Med. (2006) 166:1092–7. doi: 10.1001/archinte.166.10.1092 16717171

[29] He XY Li CB Qian J Cui HS Wu WY. Reliability and validity of a generalized anxiety disorder scale in general hospital outpatients. Shanghai Arch Psychiatry. (2010) 22:200–3. doi: 10.3969/j.issn.1002-0829.2010.04.002

[30] Correro AN 2nd Paitel ER Byers SJ Nielson KA. The role of alexithymia in memory and executive functioning across the lifespan. Cognit Emot. (2021) 35:524–39. doi: 10.1080/02699931.2019.1659232 PMC7047630 31456477

[31] Onor M Trevisiol M Spano M Aguglia E Paradiso S. Alexithymia and aging: a neuropsychological perspective. J Nerv Ment Dis. (2010) 198:891–5. doi: 10.1097/NMD.0b013e3181fe743e PMC3789519 21135641

[32] Nielson KA Meltzer MA. Modulation of long-term memory by arousal in alexithymia: the role of interpretation. Conscious Cogn. (2009) 18:786–93. doi: 10.1016/j.concog.2009.06.001 19576792

[33] Vermeulen N. Alexithymia disrupts verbal short-term memory. Cognit Emot. (2021) 35:559–68. doi: 10.1080/02699931.2019.1701418 31826706

[34] Meltzer MA Nielson KA. Memory for emotionally provocative words in alexithymia: a role for stimulus relevance. Conscious Cogn. (2010) 19:1062–8. doi: 10.1016/j.concog.2010.05.008 20538490

[35] Fernández-Fernández R Ibias J Del-Toro-Pérez C Lahera G Gasca-Salas C. Alexithymia in Parkinson’s disease: A meta-analysis. Am J geriatric Psychiatry. (2024). doi: 10.1016/j.jagp.2024.11.009 39732593

[36] Kenangil G Demir M Tur E Domac F. Alexithymia, depression, and cognition in patients with Parkinson’s disease. Acta Neurol Belg. (2023) 123:85–91. doi: 10.1007/s13760-020-01581-2 33453039

[37] Bogdanova Y Díaz-Santos M Cronin-Golomb A. Neurocognitive correlates of alexithymia in asymptomatic individuals with HIV. Neuropsychologia. (2010) 48:1295–304. doi: 10.1016/j.neuropsychologia.2009.12.033 PMC2843804 20036267

[38] O’Driscoll C Laing J Mason O. Cognitive emotion regulation strategies, alexithymia and dissociation in schizophrenia, a review and meta-analysis. Clin Psychol review. (2014) 34:482–95. doi: 10.1016/j.cpr.2014.07.002 25105273

[39] Fogley R Warman D Lysaker PH. Alexithymia in schizophrenia: associations with neurocognition and emotional distress. Psychiatry Res. (2014) 218:1–6. doi: 10.1016/j.psychres.2014.04.020 24794152

[40] Jougleux C Hennion S Outteryck O Vermersch P Zéphir H. Characterization of alexithymia in clinically isolated syndrome. Rev Neurol (Paris). (2021) 177:1145–50. doi: 10.1016/j.neurol.2021.01.017 34187691

[41] Grynberg D Luminet O Corneille O Grèzes J Berthoz S. Alexithymia in the interpersonal domain: A general deficit of empathy? Pers Individ Differences. (2010) 49:845–50. doi: 10.1016/j.paid.2010.07.013

[42] Jonason PK Krause L. The emotional deficits associated with the Dark Triad traits: Cognitive empathy, affective empathy, and alexithymia. Pers Individ Differences. (2013) 55:532–7. doi: 10.1016/j.paid.2013.04.027

[43] Santiesteban I Gibbard C Drucks H Clayton N Banissy MJ Bird G. Individuals with autism share others’ Emotions: evidence from the continuous affective rating and empathic responses (CARER) task. J Autism Dev Disord. (2021) 51:391–404. doi: 10.1007/s10803-020-04535-y 32468395

[44] Kılıç F Demirdaş A Işık Ü Akkuş M Atay İM Kuzugüdenlioğlu D. Empathy, alexithymia, and theory of mind in borderline personality disorder. J Nerv Ment Dis. (2020) 208:736–41. doi: 10.1097/nmd.0000000000001196 32520852

[45] Fujino J Yamasaki N Miyata J Kawada R Sasaki H Matsukawa N. Altered brain response to others׳ pain in major depressive disorder. J Affect Disord. (2014) 165:170–5. doi: 10.1016/j.jad.2014.04.058 24882196

[46] Banzhaf C Hoffmann F Kanske P Fan Y Walter H Spengler S. Interacting and dissociable effects of alexithymia and depression on empathy. Psychiatry Res. (2018) 270:631–8. doi: 10.1016/j.psychres.2018.10.045 30384283

[47] Cho I Cunningham TJ Daley RT Kensinger EA Gutchess A. Empathy, memory, and aging during the COVID-19 pandemic. Curr Res Ecol Soc Psychol. (2023) 4:100105. doi: 10.1016/j.cresp.2023.100105 37091210 PMC10110281

[48] Lee TH Greening SG Mather M. Encoding of goal-relevant stimuli is strengthened by emotional arousal in memory. Front Psychol. (2015) 6:1173. doi: 10.3389/fpsyg.2015.01173 26321988 PMC4530598

[49] Grabe HJ Wittfeld K Hegenscheid K Hosten N Lotze M Janowitz D. Alexithymia and brain gray matter volumes in a general population sample. Hum Brain Mapp. (2014) 35:5932–45. doi: 10.1002/hbm.22595 PMC6869686 25081815

[50] Bush G Luu P Posner MI. Cognitive and emotional influences in anterior cingulate cortex. Trends Cognit Sci. (2000) 4:215–22. doi: 10.1016/s1364-6613(00)01483-2 10827444

[51] Rolls ET. The hippocampus, ventromedial prefrontal cortex, and episodic and semantic memory. Prog Neurobiol. (2022) 217:102334. doi: 10.1016/j.pneurobio.2022.102334 35870682

[52] Clark L Chamberlain SR Sahakian BJ. Neurocognitive mechanisms in depression: implications for treatment. Annu Rev Neurosci. (2009) 32:57–74. doi: 10.1146/annurev.neuro.31.060407.125618 19400725

[53] Lupien SJ Maheu F Tu M Fiocco A Schramek TE. The effects of stress and stress hormones on human cognition: Implications for the field of brain and cognition. Brain Cogn. (2007) 65:209–37. doi: 10.1016/j.bandc.2007.02.007 17466428

[54] McEwen BS. The neurobiology of stress: from serendipity to clinical relevance. Brain Res. (2000) 886:172–89. doi: 10.1016/s0006-8993(00)02950-4 11119695

[55] Le HN Berenbaum H Raghavan C. Culture and alexithymia: mean levels, correlates, and the role of parental socialization of emotions. Emotion. (2002) 2:341–60. doi: 10.1037/1528-3542.2.4.341 12899369

[56] Grossmann I Ellsworth PC Hong YY. Culture, attention, and emotion. J Exp Psychol Gen. (2012) 141:31–6. doi: 10.1037/a0023817 21639670

[57] Sui J Zhu Y Chiu CY. Bicultural mind, self-construal, and self- and mother-reference effects: Consequences of cultural priming on recognition memory. J Exp Soc Psychol. (2007) 43:818–24. doi: 10.1016/j.jesp.2006.08.005

[58] Han S Northoff G. Culture-sensitive neural substrates of human cognition: a transcultural neuroimaging approach. Nat Rev Neurosci. (2008) 9:646–54. doi: 10.1038/nrn2456 18641669

[59] Mathur VA Harada T Chiao JY. Racial identification modulates default network activity for same and other races. Hum Brain Mapp. (2012) 33:1883–93. doi: 10.1002/hbm.21330 PMC6870292 21618667

[60] Flaskerud JH. Culture and memory. Issues Ment Health Nurs. (2013) 34:133–5. doi: 10.3109/01612840.2012.693576 23369125

[61] Hobson H Brewer R Catmur C Bird GJER. The role of language in alexithymia: moving towards a multiroute model of alexithymia. Emotion Rev. (2019) 11:247–261. doi: 10.1177/1754073919838528

[62] Camia C Desmedt O Luminet OJNI. Exploring autobiographical memory specificity and narrative emotional processing in alexithymia. Narr Inq. (2020) 30:59–79. doi: 10.1075/ni.18089.kob

[63] Hobson H Chiu EG Ravenscroft C Partridge K Bird G Demeyere N. The association between communication impairments and acquired alexithymia in chronic stroke patients. J Clin Exp Neuropsychol. (2020) 42:495–504. doi: 10.1080/13803395.2020.1770703 32576080

[64] Lee KS Catmur C Bird G. Childhood language development and alexithymia in adolescence: an 8-year longitudinal study. Dev Psychopathol. (2024) 4:1–11. doi: 10.1017/s0954579424001007 39363856

[65] Jakobson LS Pearson PM. Alexithymic traits predict the speed of classifying non-literal statements using nonverbal cues. Cognit Emot. (2021) 35:569–75. doi: 10.1080/02699931.2020.1715346 31941409

[66] Lee KS Murphy J Catmur C Bird G Hobson H. Furthering the language hypothesis of alexithymia: An integrated review and meta-analysis. Neurosci Biobehav Rev. (2022) 141:104864. doi: 10.1016/j.neubiorev.2022.104864 36087760

[67] Bosaipo NB Foss MP Young AH Juruena MF. Neuropsychological changes in melancholic and atypical depression: A systematic review. Neurosci Biobehav Rev. (2017) 73:309–25. doi: 10.1016/j.neubiorev.2016.12.014 28027956

