# Supplementary figures and images for: Immediate memory is associated with alexithymia in Chinese Han first-episode, drug-naïve major depressive disorder
Source: Front Psychiatry. 2025 Mar 26;16:1473204. doi: 10.3389/fpsyt.2025.1473204 (PMC11978825; doi:10.3389/fpsyt.2025.1473204)

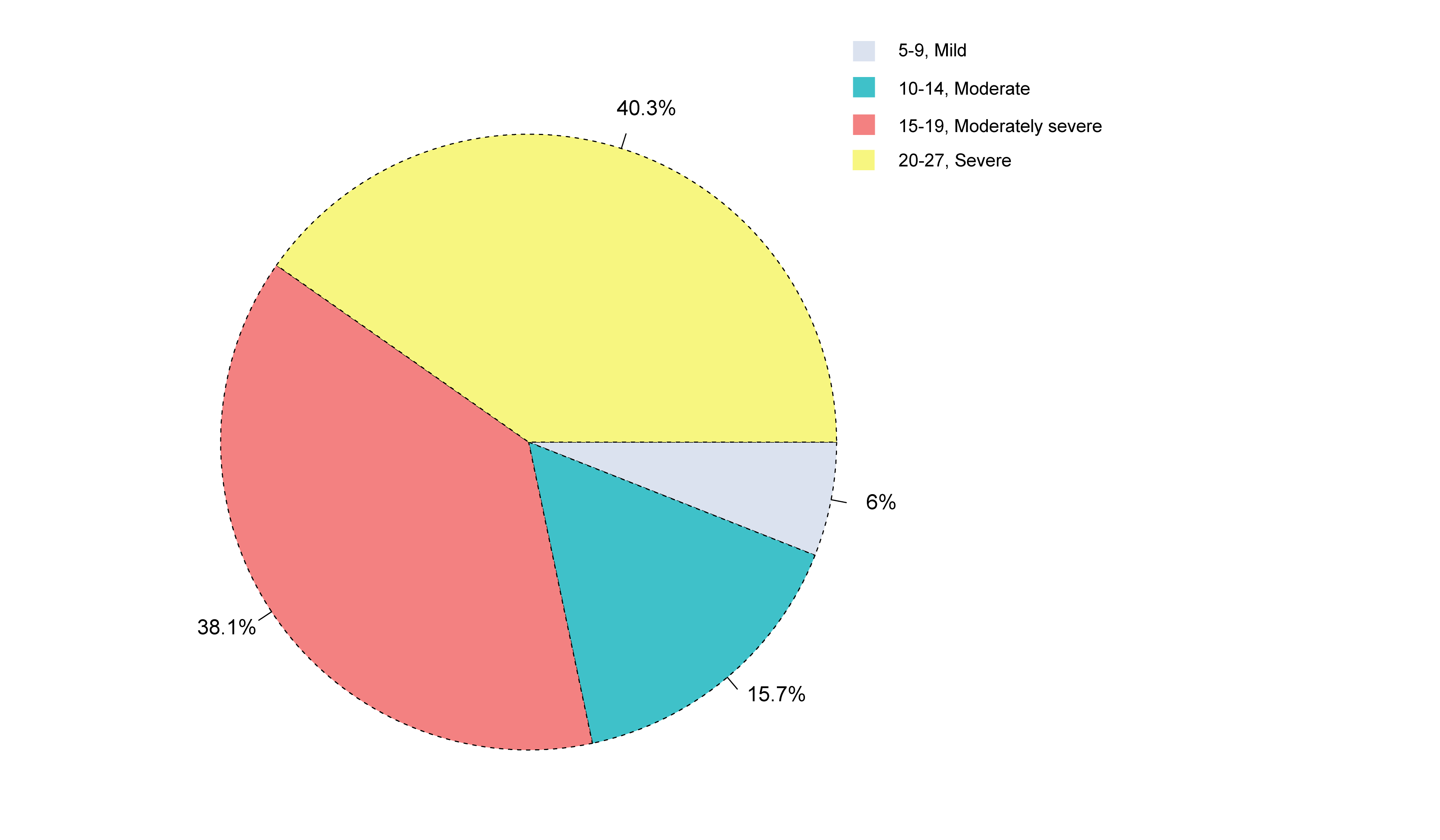

Supplement: Supplementary Figure 1 — Percentages of different depression severity levels according to PHQ-9 total score. [file Image1.tif]
